# Muscular Strength and Mortality in Women Aged 63 to 99 Years

**DOI:** 10.1001/jamanetworkopen.2025.59367

**Published:** 2026-02-13

**Authors:** Michael J. LaMonte, Eric T. Hyde, Steve Nguyen, Esmeralda Castro, Rebecca A. Seguin-Fowler, Charles B. Eaton, Connor R. Miller, Chongzhi Di, Marcia L. Stefanick, Andrea Z. LaCroix

**Affiliations:** 1 Department of Epidemiology and Environmental Health, State University of New York at Buffalo; 2 Division of Cancer Epidemiology and Genetics, National Cancer Institute, Bethesda, Maryland; 3 Herbert Wertheim School of Public Health and Human Longevity, University of California, San Diego; 4 Institute for Advancing Health Through Agriculture, Texas A&M University, College Station; 5 Departments of Family Medicine and Epidemiology, Brown University, Providence, Rhode Island; 6 Division of Public Health Sciences, Fred Hutch Cancer Center, Seattle, Washington; 7 Department of Medicine, Stanford University, Stanford, California

## Abstract

**Importance:**

Muscular strength is an important resilience marker relevant to maintaining functional independence and longevity.

**Objective:**

To examine associations between muscular strength and mortality in women aged 63 to 99 years accounting for accelerometer-measured physical activity and sedentary behavior, systemic inflammation, and other markers of aging.

**Design, Setting, and Participants:**

The Objective Physical Activity and Cardiovascular Health study was a prospective cohort study from baseline (March 2012 to April 2014) through February 19, 2023. Participants were ambulatory women aged 63 to 99 years who completed physical performance testing and 7 days of accelerometer wear.

**Exposures:**

Dominant hand grip strength,measured in kg by quartile (1: <14, 2: 14-19, 3: 19-24, and 4: >24) and time in seconds to complete 5 unassisted chair stands by quartile (standard criteria: 1: >16.7, 2: 16.6-13.7, 3: 13.6-11.2, and 4: ≤11.1).

**Main Outcome and Measure:**

All-cause mortality.

**Results:**

The present study included 5472 women (mean [SD] age, 78.7 [6.7] years; 1851 [33.8%] Black; 915 [16.7%] Hispanic/Latina; 2706 [49.5%] White) followed up for a mean (SD) of 8.4 (2.4) years. There were 1964 deaths during the study period. Controlling for age and sociodemographic, lifestyle, and clinical factors, significant inverse trends in mortality were evident across quartiles 2 through 4 of grip strength (quartile 2: hazard ratio [HR], 0.94; 95% CI, 0.85-1.06; quartile 3: HR, 0.85; 95% CI, 0.75-0.97; quartile 4: HR, 0.67; 95% CI, 0.58-0.78; *P *for trend < .001) and chair stand time (quartile 2: HR, 0.79; 95% CI, 0.69-0.88; quartile 3: HR, 0.76; 95% CI, 0.67-0.87; quartile 4: HR, 0.63; 95% CI, 0.54-0.73; *P *for trend < .001). Further controlling simultaneously for sedentary time and moderate to vigorous physical activity attenuated associations (grip strength: quartile 2: HR, 0.95; 95% CI, 0.86-1.07; quartile 3: HR, 0.87; 95% CI, 0.76-0.99; quartile 4: HR, 0.70; 95% CI, 0.61-0.82; *P *for trend < .001; chair stands: quartile 2: HR, 0.82; 95% CI, 0.73-0.92; quartile 3: HR, 0.82; 95% CI, 0.71-0.93; quartile 4: HR, 0.69; 95% CI, 0.59-0.79; *P *for trend < .001). Similar inverse associations were observed when controlling for walking speed and the inflammatory marker C-reactive protein. Magnitudes of association did not differ across subgroups defined by age, race and ethnicity, body mass index, moderate-to-vigorous physical activity, sedentary time, or timed walk.

**Conclusions and Relevance:**

In this study of ambulatory older women, greater muscular strength was associated with lower mortality even when controlling for accelerometer-measured PA and sedentary time, walking speed, and systemic inflammation. These findings suggest that assessing strength and promoting its maintenance are instrumental for optimal aging.

## Key Points

**Question:**

Is muscular strength associated with mortality in older women after controlling for aerobic activity, sedentary time, and fitness level?

**Findings:**

In this cohort study of 5472 women aged 63 to 99 years, 2 common strength tests were associated with significantly lower mortality risk after controlling for sociodemographic and clinical characteristics, accelerometer-measured physical activity and sedentary behavior, and timed walk. Muscle strength was associated with lower mortality even in women not meeting guideline-recommended activity levels.

**Meaning:**

These findings suggest that strength can be easily assessed in the clinical setting, and promoting its maintenance could play a key role in optimal aging.

## Teaser

This cohort study evaluates associations between muscular strength and mortality in women aged 63 to 99 years accounting for accelerometer-measured physical activity and sedentary behavior, systemic inflammation, and other markers of aging.

## Introduction

The Physical Activity Guidelines for Americans, 2nd edition (2018), and a 2024 American Heart Association Scientific Statement provide formal recommendations for participation in skeletal muscle strengthening activities at least 2 days per week to achieve systemic and functional health benefits.^1,2^ Among middle-aged and older adults, studies have shown inverse associations between muscular strength and mortality.^2,3,4,5^ Meta-analyses of prospective studies showed all-cause mortality risk was 15% lower for those who self-reported any compared with no strengthening activity,^5^ and was 8% lower for each 5 kg greater grip strength.^6^ An association between muscle strength and mortality could be influenced by one’s level of aerobic physical activity and sedentary behavior. Previous studies on muscular strength and mortality have controlled for self-reported aerobic physical activity levels; however, self-report assessment does not completely capture all sources of daily movement, nor does it accurately assess sedentary time, particularly in older women.^7^ Likewise, cardiorespiratory fitness is strongly associated with mortality in older adults,^8^ but few studies on muscle strength and mortality have addressed this relevant source of confounding. Aging-related inflammation causes mitcohondrial dysfunction and impairs skeletal muscle excitation-contraction coupling.^9^ Skeletal muscle strength declines rapidly during adulthood,^10^ yet it is integral to maintaining functional independence,^11^ averting hospitalizations,^12^ and improving quality of life.^13^

The primary objective of this study was to determine associations with all-cause mortality for 2 common measures of muscular strength while controlling for accelerometer-measured physical activity and sedentary time, walking speed (a proxy for cardiorespiratory fitness), and systemic inflammation in a cohort of ambulatory women aged 63 to 99 years.

## Methods

### Study Population

Participants were enrolled in the Objective Physical Activity and Cardiovascular Health in Older Women (OPACH) study which is ancillary to the Women’s Health Initiative (WHI). Details about OPACH^14^ and WHI^15^ have been published. Study protocols were approved by institutional review boards at the clinical centers and participants provided written informed consent before data collection. This manuscript followed the Strengthening the Reporting of Observational Studies in Epidemiology (STROBE) reporting guidelines for cohort studies.

During 2012 to 2014, in-home examinations were completed in 7875 WHI participants. Fasting blood draw, body weight and height, resting blood pressure, grip strength, and the short physical performance battery (SPPB)^16^ were collected. Of these women, 7048 (90%) further consented into OPACH and received a triaxial accelerometer (GT3X+; ActiGraph) and instructions for wear on their hip, 24 hours per day for 7 consecutive days. The OPACH laboratory calibration study determined vector magnitude (counts per 15 seconds) cut points defining sedentary (<19), light (19-518), and moderate to vigorous physical activity (MVPA; ≥519) intensity activities.^17^

### Muscular Strength Assessments

Dominant hand grip strength was assessed using a dynamometer (Jamar, Lafayette Instruments) in participants who were without severe upper limb disability (eg, prior stroke disability or functionally limiting arthritis). Testing was done in the seated position with the back supported, the elbow flexed to 90 degrees, and the forearm parallel to the floor. After a practice trial at half effort, two trials at maximal effort separated by 60 seconds were completed. The highest of the 2 trials was analyzed here, defined as quartiles (1: <14, 2: 14-19, 3: 20-24, 4: >24 kg).

Chair stand performance was assessed as part of the SPPB^16^ with the back of the chair against the wall for stability and participant’s arms crossed in front of their chest. Participants attempted a single repetition to determine ability and safety, then performed 5 consecutive sit-to-stands without assistance. Time to completion in seconds was recorded for analysis, and categorized according to criteria in Established Populations for the Epidemiologic Study of the Elderly (≥16.7, 16.6-13.7, 13.6-11.2, or ≤11.1 seconds).^16^

### Timed Walk Test

Participants performed a timed 2.5-m (8 ft) walk at usual pace during the SPPB. The walk course was on a flat surface in the home marked by a 2.5-m line between 2 cones. Participants wore comfortable walking shoes, were permitted to use walking aids, and could stop to rest during the walk. The total time to complete the walk, including rests, was recorded for 2 trials separated by 3 minutes. Here, we use walk time as a proxy of cardiorespiratory fitness.^18^

### Mortality

Mortality surveillance was completed annually through mailed health update questionnaires augmented by National Death Index searches, proxy queries, obituaries, and hospital records to identify deaths.^19^ The mortality end point in the present analysis was deaths from all causes for which ascertainment is more than 99% complete in the WHI.

### Covariates

Race and ethnicity, education, age at menopause, smoking status, alcohol intake, self-rated general health, and walking aid use were self-reported. Participants self-identified race and ethnicity from questionnaire categories (Black, Hispanic/Latina, and White). We include this covariate to characterize the study cohort and because physical performance and mortality vary with race and ethnicity. Body weight (kg), standing height (cm), and resting systolic and diastolic blood pressure (mm Hg) were measured during the in-home exam. Lean body mass (LBM) was estimated using an equation developed previously in 4519 women aged 20 to 80 years participating in the National Health and Nutrition Examination Study (1999-2006) wherein dual energy x-ray absorptiometry (DXA) was the criterion.^20^ The equation: LBM (kg) = (age × −0.039) + (HT × 0.186) +(WT × 0.383) + (waist × −0.043) + (race) – 10.683, where age is in years, height in centimeters, weight in kilograms, waist in centimeters, and race coded 0 for non-Hispanic White, 1.085 for non-Hispanic Black, and −0.059 for Hispanic women. Estimation of LBM has an *R*^2^ = 0.91 (standard error of estimation, 2.6 kg) and the difference between measured and estimated was 0.07 kg (*P* = .42).^20^ Presence of 11 comorbid conditions (coronary heart disease, stroke, cancer, diabetes, hip fracture, osteoarthritis, depression, chronic obstructive pulmonary disease, cognitive impairment, sensory impairment, and frequent falls during the past year) were summarized as an ordinal variable from 0 to 11. Serum high-sensitivity C-reactive protein (CRP; mg/dL) was available in 4414 participants^21^ and was evaluated to determine the extent that systemic inflammation is associated with variation between muscular strength and mortality in aging women.

### Statistical Analysis

The muscle strength variables were analyzed both categorically (defined previously) and continuously (per 1 SD greater grip strength or faster chair stands) to maximize comparisons with published data. Analysis of variance and χ^2^ tests were used to evaluate baseline characteristics over muscle strength categories. Time-to-event was accrued from the date of muscle strength assessment to the date of death from any cause, loss to follow-up (<2%), or February 19, 2023, whichever came first. Cox proportional hazards regression was used to estimate hazard ratios (HR) and 95% CIs for mortality according to muscle strength. The proportionality assumption was assessed using Schoenfeld residuals; no appreciable violations were noted. Progressive model adjustments included age (years) and race and ethnicity (model 1); then additionally, education (<high school, some college, or ≥college), body weight, current smoking (no or yes), alcohol (drinks per wk), age at menopause (years), systolic and diastolic blood pressure, self-rated general health (excellent or very good, good, or fair or poor), walking aid use (no or yes), and comorbidity score (0-11) (model 2); additionally to model 2, accelerometer-measured total sedentary time (hours per day) and MVPA (minutes per day) (model 3); and again additionally to model 2, accelerometer-measured total sedentary time and 2.5-m walk time (minutes). Trend tests were conducted with a Wald test on strength categories assigned their median values. Consistency of associations was explored using stratification across cohort subgroups defined by age (<80 or ≥80 years), race and ethnicity (Black, Hispanic/Latina, or White), BMI (<18.5, 18.5-24.9, or ≥25.0; calculated as weight in kilograms divided by height in meters squared), MVPA (median split), sedentary time (median split), and 2.5-m walk time (median split). Interaction tests using model cross-product terms at α = .05 were conducted in an exploratory manner. To evaluate the likelihood of reverse causation bias we completed a sensitivity analysis that excluded women who died during the first 3 and 5 years of follow-up. There is published evidence that in older adults measures of grip strength are associated similarly with mortality outcomes whether defined as absolute (kg strength) or relative to body mass (kg strength per kg body mass) or LBM (kg per kg LBM).^22^ Thus, we conducted a sensitivity analysis using relative grip strength as the exposure. Analyses were completed using SAS version 9.4 (SAS Institute), and *P* values are 2-sided for significance tests conducted at α = .05.

## Results

### Study Population

Overall, the cohort’s mean (SD) age was 78.7 (6.7) years and included 1851 (33.8%) Black, 915 (16.7%) Hispanic, and 2706 (49.5%) White women (Table 1). A total of 4349 (79.4%) had at least some college education, 135 (2.5%) were current smokers, 1595 (29%) reported walking aid use, 2543 (46.5%) rated their general health as very good to excellent, and 4529 (82.7%) had at least 1 comorbid condition. The mean (SD) height, weight, BMI, and estimated LBM were 159.7 (7.2) cm, 71.7 (15.1) kg, 28.1 (5.6), and 39.9 (6.3) kg, respectively. Mean (SD) systolic and diastolic BP were 125.7 (13.8) and 72.6 (8.5) mm Hg, RAND-36 physical function score was 68.6 (25.9), light PA was 284.4 (78.9) minutes per day and MVPA was 49.7 (34.4) minutes per day, sedentary time was 9.2 (1.7) hours per day, grip strength was 19.3 (7.1) kg, and chair stand time was 15.4 (5.8) seconds. With the exception of systolic BP and CRP, each baseline characteristic was significantly associated with quartiles of grip strength. eTable 1 in Supplement 1 provides baseline characteristics according to vital status for which statistically significant differences were present between decedents and survivors for all characteristics.

**Table 1.** Baseline Characteristics for the Overall Cohort and by Quartiles of Grip Strength

Characteristic	Participants, No. (%)					*P* value^a^
	Overall (n = 5472)	Grip strength quartile, kg				
		<14 (n = 1085)	14-19 (n = 1632)	20-24 (n = 1280)	>24 (n = 1475)	
Age, mean (SD), y	78.7 (6.6)	81.8 (6.2)	79.9 (6.4)	77.8 (6.5)	75.6 (6.0)	<.001
Race and ethnicity						
Black	1851 (33.8)	231 (21.3)	447 (27.4)	449 (35.1)	724 (49.1)	<.001
Hispanic/Latina	915 (16.7)	156 (14.4)	288 (17.7)	244 (19.1)	227 (15.4)	
White	2706 (49.5)	698 (64.3)	897 (54.9)	587 (45.9)	524 (35.5)	
Education						
≤High school	1123 (20.5)	255 (23.5)	352 (21.6)	251 (19.6)	265 (17.9)	.002
Some college	2121 (38.8)	416 (38.3)	652 (39.9)	500 (39.1)	553 (37.5)	
≥College graduate	2228 (40.7)	414 (38.2)	628 (38.5)	529 (41.3)	657 (44.5)	
Current smoker	135 (2.5)	13 (1.2)	32 (1.9)	40 (3.1)	50 (3.4)	.001
Use walking aid	1595 (29.2)	446 (41.1)	525 (32.2)	325 (25.4)	299 (20.3)	<.001
Self-rated health						
Excellent or very good	2543 (46.5)	420 (38.7)	721 (44.2)	646 (50.5)	756 (51.2)	<.001
Good	2462 (44.9)	544 (50.1)	743 (45.5)	535 (41.8)	640 (43.4)	
Fair or poor	467 (8.5)	121 (11.2)	168 (10.3)	99 (7.7)	79 (5.4)	
Age at menopause, mean (SD), y	48.2 (6.3)	48.3 (6.4)	48.4 (6.3)	47.9 (6.2)	47.9 (6.4)	.09
Height, mean (SD), cm	159.7 (7.2)	157.8 (7.0)	158.5 (7.1)	160.0 (7.1)	162.1 (7.1)	<.001
Weight, mean (SD), kg	71.7 (15.1)	69.2 (15.1)	69.8 (14.8)	72.2 (15.4)	76.1 (16.1)	<.001
BMI, mean (SD)^b^	28.1 (5.6)	27.8 (5.7)	27.8 (5.6)	28.2 (5.7)	28.9 (5.9)	<.001
LBM, mean (SD), kg	39.9 (6.3)	38.3 (6.1)	38.9 (6.0)	40.2 (6.2)	42.3 (6.4)	<.001
Systolic BP, mean (SD), mm Hg	125.7 (13.8)	125.9 (14.8)	126.2 (14.2)	125.6 (14.4)	125.6 (13.8)	.58
Diastolic BP, mean (SD) mm Hg	72.6 (8.5)	71.9 (8.9)	72.3 (8.9)	72.8 (8.6)	73.3 (8.6)	<.001
Comorbidities, mean (SD)^c^	1.6 (1.3)	1.9 (1.4)	1.7 (1.2)	1.5 (1.2)	1.4 (1.2)	<.001
None	943 (17.2)	120 (11.1)	221 (13.5)	242 (18.9)	360 (24.4)	<.001
1-2	3395 (62.1)	643 (59.3)	1042 (63.9)	814 (63.6)	896 (60.8)	
≥3	1134 (20.7)	322 (29.6)	369 (22.6)	224 (17.5)	219 (14.8)	
Log CRP, mean (SD), mg/dL^d^	0.62 (1.1)	0.63 (1.1)	0.59 (1.0)	0.64 (1.0)	0.64 (1.1)	.48
Alcohol, mean (SD), drinks/wk	0.9 (0.8)	0.9 (0.8)	0.9 (0.8)	0.9 (0.8)	1.0 (0.8)	.01
Physical function, mean (SD)^e^	68.6 (25.9)	59.6 (27.2)	66.6 (25.5)	72.2 (24.4)	75.9 (23.5)	<.001
Light PA, mean (SD), min/d	284.4 (78.9)	273.2 (79.6)	281.8 (78.7)	290.8 (78.9)	293.7 (76.2)	<.001
MVPA, mean (SD), min/d	49.7 (34.4)	40.7 (30.2)	46.6 (32.4)	52.6 (34.4)	59.9 (37.1)	<.001
Sedentary time, mean (SD), h/d	9.2 (1.7)	9.4 (1.6)	9.3 (1.7)	9.1 (1.7)	9.0 (1.7)	<.001
2.5-m Walk time, mean (SD), min	7.9 (6.2)	9.3 (7.6)	7.6 (5.5)	7.8 (6.9)	6.8 (4.2)	<.001
Grip strength, mean (SD), kg	19.3 (7.1)	9.8 (2.7)	16.4 (1.7)	20.9 (1.0)	28.2 (4.5)	<.001
Chair stand time, mean (SD), s	15.4 (5.8)	16.3 (6.4)	15.6 (5.3)	15.3 (6.7)	14.5 (5.1)	<.001

Abbreviations: BMI, body mass index; BP, blood pressure; CRP, C-reactive protein; LBM, lean body mass; MVPA, moderate to vigorous physical activity; PA, physical activity.

^a^ Trend across grip strength quartiles based on analysis of variance (continuous) or χ^2^ (categorical).

^b^ Calculated as weight in kilograms divided by height in meters squared.

^c^ Comorbidities defined as heart disease, stroke, cancer, diabetes, hip fracture, osteoarthritis, depression, chronic obstructive pulmonary disease, cognitive impairment, sensory impairment, and frequent falls in past year.

^d^ 4414 participants.

^e^ RAND-36 physical function score, range 0-100, higher score reflects better functional status.

eTable 2 in Supplement 1 provides unadjusted Spearman correlations among selected variables. Grip strength and chair stand time were inversely correlated with each other (*r* = −0.13) and positively correlated with body weight (*r* = 0.19 and *r* = 0.15, respectively).

**Figure 1.**
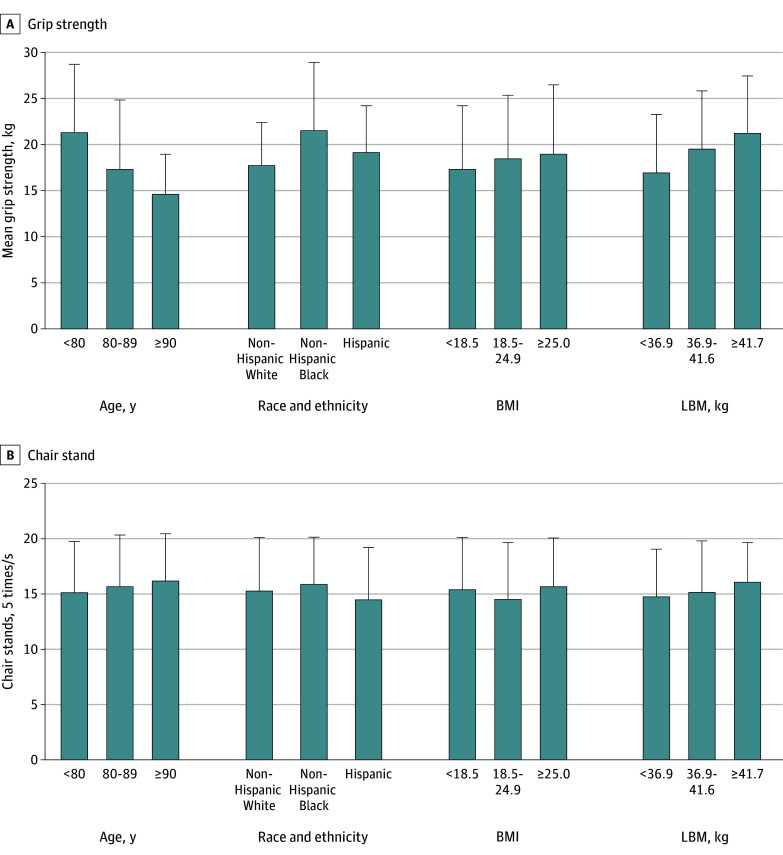
Mean Grip Strength and Chair Stand Time According to Age, Race and Ethnicity, Body Mass Index (BMI), and Lean Body Mass (LBM) Error bars indicate SDs. See Table 1 for age, race and ethnicity, and BMI group sizes. LBM groups included 1872, 1664, and 1936 women, respectively. BMI is calculated as weight in kilograms divided by height in meters squared.

Figure 1 shows unadjusted mean grip strength and chair stand speed according to categories of age, race and ethnicity, BMI, and estimated LBM. Grip strength was incrementally lower with greater age, variable over race and ethnicity, and incrementally higher with greater BMI or LBM. Chair stand time was slower with older ages, was variable over race and ethnicity and BMI, and was slower with greater LBM.

### Muscular Strength and Mortality

During a mean (SD) of 8.3 (2.4) years of follow-up, 1964 (35.8%) women died from all causes. Kaplan-Meier plots demonstrated statistically significant differences in mortality over follow-up time according to categories of grip strength and chair stand time (eFigure in Supplement 1). An inverse gradient in unadjusted death rates (per 1000 person-years) existed across incremental quartiles of grip strength (67.2, 52.3, 37.4, 23.5, respectively) and faster chair stand times (60.2, 39.1, 33.9, 26.5, respectively) (Table 2). Controlling for age and race and ethnicity, lower mortality HRs were evident with greater grip strength across quartiles (HR, 0.90; 95% CI, 0.81-1.00; HR, 0.78; 95% CI, 0.68-0.88; HR, 0.60; 95% CI, 0.52-0.69, respectively; *P *for trend < .001) and faster chair stand time across quartiles (HR, 0.69; 95% CI, 0.62-0.78; HR, 0.64; 95% CI, 0.57-0.74; HR, 0.49; 95% CI, 0.43-0.57, respectively; *P *for trend < .001). The HR for a 1 SD unit greater grip strength (7 kg) and faster chair stand time (6 seconds) was 0.85 (95% CI, 0.81-0.89) and 0.91 (95% CI, 0.88-0.94), respectively. Significant inverse mortality trends over quartiles of grip strength (HR, 0.94; 95% CI, 0.85-1.06; HR, 0.85; 95% CI, 0.75-0.97; HR, 0.67; 95% CI, 0.58-0.78, respectively; *P *for trend < .001) and chair stand times (HR 0.79; 95% CI, 0.69-0.88; HR, 0.76; 95% CI, 0.67-0.87; HR, 0.63; 95% CI, 0.54-0.73, respectively; *P *for trend < .001) persisted with further adjustment for sociodemographic factors, lifestyle behaviors, and number of comorbidities. The HR per 1 SD unit attenuated remaining significance for grip strength (HR, 0.88; 95% CI, 0.84-0.93) but not for chair stand time (HR, 0.96; 95% CI, 0.93-1.01). When sedentary time and MVPA were simultaneously added to the model, significant inverse trends in mortality risk remained for categories of both strength measures. Substituting total physical activity for MVPA did not materially change the significant inverse associations across muscular strength categories. When sedentary time and 2.5-m walk time were simultaneously included in the model, muscle strength remained significantly inversely associated with mortality (Table 2 model 4).

**Table 2.** Hazard Ratios (HR) and 95% CIs for All-Cause Mortality According to Muscular Strength Levels (N = 5472)

Measure and model^a^	HR (95% CI)				*P* value for trend	Per 1 SD
	Quartile 1	Quartile 2	Quartile 3	Quartile 4		
**Grip strength**						
Weight, kg	<14	14-19	20-24	>24		
Total No.	1085	1632	1280	1475	NA	NA
Deaths, No. (mortality rate)^b^	554 (67.2)	695 (52.3)	405 (37.4)	310 (23.5)	NA	NA
Model 1	1 [Reference]	0.90 (0.81-1.00)	0.78 (0.68-0.88)	0.60 (0.52-0.69)	<.001	0.85 (0.81-0.89)
Model 2	1 [Reference]	0.94 (0.85-1.06)	0.85 (0.75-0.97)	0.67 (0.58-0.78)		0.88 (0.84-0.93)
Model 3	1 [Reference]	0.95 (0.86-1.07)	0.87 (0.76-0.99)	0.70 (0.61-0.82)		0.89 (0.85-0.94)
Model 4	1 [Reference]	0.94 (0.84-1.06)	0.83 (0.73-0.96)	0.65 (0.56-0.76)		0.87 (0.83-0.92)
**Chair stand time**						
Time, s	≥16.7	16.6-13.7	13.6-11.2	≤11.1	NA	NA
Total No.	2076	1304	1077	1015		
Deaths, No. (mortality rate)^b^	975 (60.2)	435 (39.1)	316 (33.9)	238 (26.5)		
Model 1	1 [Reference]	0.69 (0.62-0.78)	0.64 (0.57-0.74)	0.49 (0.43-0.57)	<.001	0.91 (0.88-0.94)
Model 2	1 [Reference]	0.79 (0.69-0.88)	0.76 (0.67-0.87)	0.63 (0.54-0.73)		0.96 (0.93-1.01)
Model 3	1 [Reference]	0.82 (0.73-0.92)	0.82 (0.71-0.93)	0.69 (0.59-0.79)		0.98 (0.94-1.02)
Model 4	1 [Reference]	0.82 (0.73-0.93)	0.79 (0.69-0.91)	0.66 (0.57-0.78)		0.98 (0.94-1.02)

Abbreviation: NA, not applicable.

^a^ Model 1 included age and race and ethnicity. Model 2 included model 1 covariates plus education, body weight, systolic and diastolic blood pressure, number of comorbidities, alcohol, age at menopause, use of walking aids, smoking, and self-rated general health. Model 3 included model 2 covariates plus accelerometer-measured sedentary time and moderate-to-vigorous physical activity. Model 4 included model 2 covariates plus accelerometer-measured sedentary time and 2.5-m walk time.

^b^ Crude rate per 1000 person-years.

**Figure 2.**
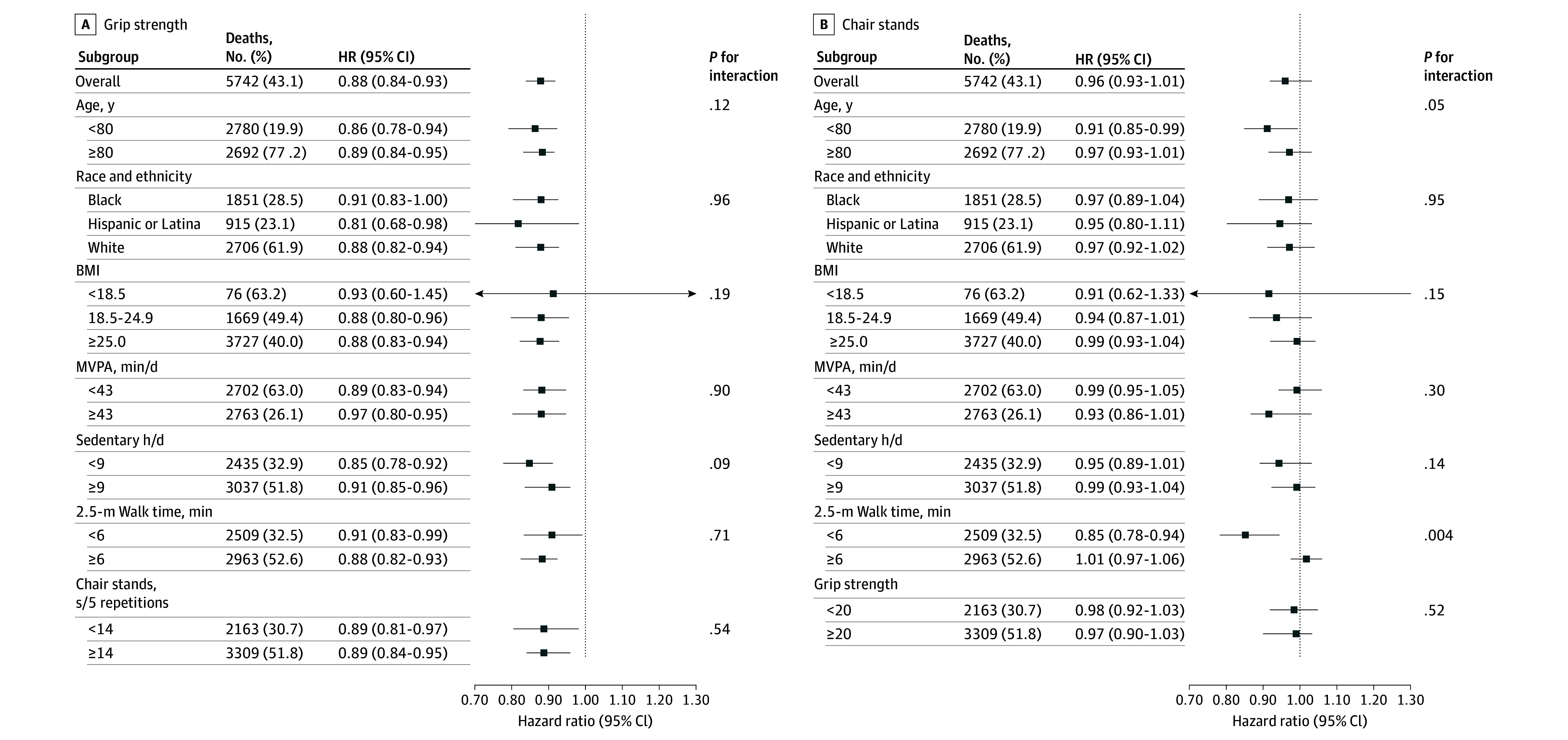
Associations of Grip Strength and Chair Stands With All-Cause Mortality According to Cohort Subgroups Death rate is per 1000 person-years. Hazard ratios and 95% CIs are per 1 SD unit greater grip strength or faster chair stands. Model included age, race and ethnicity, education, body weight, systolic and diastolic blood pressure, number of comorbidities, alcohol, age at menopause, use of walking aids, smoking, and self-rated general health. *P* interaction from a Wald test on model cross-product terms for grip strength (or chair stand) and the subgroup of interest. BMI indicates body mass index (calculated as weight in kilograms divided by height in meters squared); MVPA, moderate to vigorous physical activity.

Despite large differences in subgroup mortality rates, HRs for all-cause mortality were of consistent direction and magnitude over subgroup strata for both grip strength and chair stands (Figure 2). Within most subgroups, grip strength was significantly associated with mortality but chair stands were not. When MVPA was additionally categorized as meeting (≥150 minutes per week; 1244 deaths) or not meeting (<150 minutes per week; 720 deaths) guideline recommended levels, grip strength was significantly inversely associated with mortality risk in women meeting (per 1 SD: HR, 0.88; 95% CI, 0.83-0.94) and in women not achieving (per 1 SD: HR, 0.90; 95% CI, 0.82-0.98) recommended levels of aerobic activity. Chair stand time was not associated with mortality in either group.

There was a slight attenuation of the inverse association when grip strength quartiles were defined relative to body weight (kg per kg body weight), (HR, 0.91; 95% CI, 0.81-1.02; HR, 0.88; 95% CI, 0.77-0.99; HR, 0.81; 95% CI, 0.70-0.92, respectively; *P* for trend = .01; per 1 SD: (HR, 0.89; 95% CI, 0.85-0.94). Results were similar when strength was relative to estimated LBM (kg per kg LBM) (HR, 0.94; 95% CI, 0.83-1.05; HR, 0.85; 95% CI, 0.75-0.96; HR, 0.75; 95% CI, 0.66-0.86, respectively; *P *for trend < .001; per 1 SD: HR, 0.89; 95% CI, 0.85-0.93).

When CRP was added to Cox regression model 2 (4414 participants; 1592 deaths) significant inverse associations remained across quartiles of absolute grip strength (HR, 0.96; 95% CI, 0.85-1.09; HR, 0.88; 95% CI, 0.76-1.00; HR, 0.66; 95% CI, 0.56-0.78, respectively; *P *for trend < .001; per 1 SD: HR, 0.88; 95% CI, 0.84-0.93) and chair stand time (HR, 0.80; 95% CI, 0.70-0.91; HR, 0.75; 95% CI, 0.65-0.87; HR, 0.64; 95% CI, 0.54-0.75, respectively; *P *for trend < .001; per 1 SD: HR, 0.98; 95% CI, 0.93-1.03). These associations were only modestly attenuated compared with primary results (model 2).

Sensitivity analyses were completed to understand robustness of primary results. After excluding women who died during the first 3 and 5 years, associations with mortality for grip strength and chair stand time were comparable with primary findings (eTable 3 in Supplement 1). To further discern muscle strength from aerobic physical activity, we restricted analysis to only women with MVPA less than 10 minutes per day (368 participants; 265 deaths) and the inverse association remained evident for grip strength (per 1 SD: HR, 0.86; 95% CI, 0.74-1.01; *P* = .07) but not chair stands (per 1 SD: HR, 1.09; 95% CI, 0.96-1.22; *P* = .18), though smaller sample sizes were limiting. To probe further the association of functional status with muscle strength and mortality, we stratified associations by walking device use (1595 participants; 840 deaths) or no use (3877 participants; 1124 deaths). Grip strength was inversely associated with mortality in walking device users (per 1 SD: HR, 0.87; 95% CI, 0.80-0.94) and nonusers (HR, 0.89; 95% CI, 0.83-0.95), whereas chair stands were associated with mortality only among nonusers (HR, 0.91; 95% CI, 0.86-0.96) and not users (HR, 1.04; 95% CI, 0.98-1.11). To better understand associations of mortality with muscle strength (model 2 covariates including age) in the context of aging, stratification across broader age categories are given in eTable 4 in Supplement 1. Findings were consistent with those in Figure 2 based on binary age groups, though extreme age sample sizes were limited. We also repeated the Cox regression analysis (model 2 covariates; eTable 5 in Supplement 1) using age as the time scale. Results were similar to those where time on study was the scale.

## Discussion

By 2050, women aged 75 years and older will be the largest age subgroup in the US.^23^ Physical activity is an established component in optimal aging.^24^ Historically, aerobic physical activity has been the focus for health and longevity.^25,26,27^ Recent national guidelines have included recommendations promoting muscular strength, ostensibly to extend benefits conferred by regular aerobic activity.^1,2^ Few studies on strength and health outcomes have accounted for contemporaneously measured aerobic physical activity, sedentary time, or cardiorespiratory fitness; even fewer have focused on older women. In the present study, 2 measures of muscular strength that can easily be assessed in clinical settings were significantly associated with lower mortality in women aged 63 to 99 years. The inverse associations were robust to controlling for several relevant covariates including age, body weight, smoking, walking aid use, physical functioning score, number of comorbidities, and systemic inflammation. The inverse association was evident whether grip strength was modeled absolute or relative to total body weight or estimated LBM. Moreover, the significant inverse associations were robust to simultaneous adjustment for accelerometer-measured sedentary time and MVPA, as well as for 2.5-m walk time as a proxy of cardiorespiratory fitness. The direction and strength of associations between muscular strength and mortality were consistent after excluding early deaths from analyses and when using age as the time scale in the regression model. Notably, muscular strength was associated with lower mortality even in women who did not meet guideline-recommended amounts of aerobic physical activity.

The correlation between grip strength and chair stand time was relatively small (*r* = −0.13) with less than 2% shared variation. This suggests that the 2 measures are largely assessing different constructs. Additionally, the association between grip strength and mortality attenuated little when adjusted for health and clinical factors, whereas the association for chair stands attenuated almost to the null. This suggests that chair stand time may be a more general biomarker of aging-related health status including fatiguability,^28^ whereas grip strength may be a biomarker better reflective of skeletal muscle strength output. Grip strength could also be more representative of upper body strength that is used more routinely in activities as aging occurs as compared with lower body strength during chair stands.

We were able to account for objectively measured free-living physical activity and sedentary time. Previous studies have controlled for self-reported physical activity habits^22,29,30,31,32,33^ and sedentary time.^33^ Self-report assessments provide an incomplete quantification of daily physical activity and sedentary time in older adults.^7^ Thus, previous studies on strength and mortality might have suffered from residual confounding by aerobic activity or sedentary time. Here, when the multivariable Cox regression models simultaneously included sedentary time and either MVPA or total physical activity, a significant inverse association for muscle strength was observed. Even when the study sample was restricted to women with very little MVPA (<10 minutes per day) there was an association between strength and mortality. The significantly lower mortality risk associated with greater grip strength in participants whose physical activity was below guideline recommended levels (<150 minutes per week) and in those who used a walking device is important because some older women may not be able to engage in regular aerobic activity but still might enhance health and longevity through maintenance of muscular strength levels. Individuals who lack the muscular strength to initiate movement also are less likely able to engage in health-enhancing aerobic activities.

Cardiorespiratory fitness is strongly associated with mortality^34^ and is a hallmark resiliency factor.^35^ Few previous studies on muscular strength and mortality have accounted for the influence of cardiorespiratory fitness.^36,37^ Among 230 670 women aged 38 to 73 years in the UK Biobank, the inverse association between grip strength and all-cause mortality attenuated to nonsignificant when self-reported walking speed (proxy of cardiorespiratory fitness) was added to the multivariable model.^36^ In our study on women aged 63 to 99 years, when timed walk (a proxy of fitness) was included in the multivariable Cox regression model, mortality risk was lower when comparing the highest and lowest strength categories (Table 2, model 4). Maintaining muscular strength could contribute to optimal aging through pathways distinct from cardiorespiratory fitness. Because lower mortality risk was observed in regression models that simultaneously included accelerometer-measured sedentary and physical activity or walk time, walking aid use, physical functioning score, self-rated general health, and number of comorbidities, our finding is less likely due to reverse causation providing a clearer measure of association than reported in previous epidemiologic studies on older adults.

Chronic systemic inflammation leads to a maladaptive immune response that accelerates loss of skeletal muscle mass and function^38,39^ and increases mortality^40^ in older adults. In our study, baseline CRP concentrations were significantly higher in decedents compared with survivors (eTable 1 in Supplement 1), and were inversely associated with muscular strength measures. The association between muscular strength and mortality attenuated only modestly when adjusted for CRP. Likewise, a previous study reported a small attenuation (approximately 3%) by CRP when comparing category extremes.^30^ This does not negate the established role inflammation has in reducing muscle function and increasing mortality risk in aging. However, the influence muscle strength has on mortality seemingly involves mechanisms beyond inflammation. Future studies evaluating other biomarkers in the inflammatory cascade will provide clarification.

### Limitations

While grip strength and chair stands are feasible assessments in epidemiological cohort studies, more elaborate measures such as isokinetic tests^29^ and upper and lower body maximal weight lifted^37^ could provide a more comprehensive measure of total body strength. We did not have imaging to quantify body composition, particularly LBM, which we estimated. However, results from the Health Aging and Body Composition study^29^ showed a significant inverse association between isokinetic knee extensor strength and mortality when controlling for cross-sectional muscle area measured by computed tomography and body fat mass and LBM measured by DXA. This suggests that muscle quality is a more relevant aspect of optimal aging than muscle mass, per se. We relied on 2.5-m walk time as a proxy for fitness. Walking speed is strongly correlated with treadmill exercise time^18^ and maximal oxygen uptake,^36^ the reference standard measure of fitness, which enhances confidence in our result. Nutritional status is important for maintaining skeletal muscle mass and function in aging, and is strongly associated with mortality in older adults. Information on nutrition status was not systematically collected in OPACH women. A previous study showed an inverse association between grip strength and mortality in older women that was independent of dietary intake^30^ and unintentional weight loss.^30^ In a sensitivity analysis we excluded deaths during the first 3 and 5 years of follow-up, which might include undernourished women or those with cachexia, and results were comparable with the primary findings. Because OPACH is ancillary to the national Women’s Health Initiative, which focused on postmenopausal women, our results do not necessarily generalize to men or to women in earlier life stages.

## Conclusions

In this cohort study of ambulatory women aged 63 to 99 years, higher skeletal muscle strength was associated with significantly lower all-cause mortality. This association was evident after controlling for several strong mortality risk factors and in women not meeting aerobic activity guidelines. Our finding supports current national recommendations that promote participation in muscle strengthening activity for optimal aging and longevity. To improve guideline recommendations, future research should better characterize the type and amounts of muscular strengthening activity associated with more specific outcomes across the health span.

## Notes

Supplement 1. eTable 1. Baseline Characteristics by Vital Status eTable 2. Spearman Correlation Coefficients eTable 3. Results When Excluding Early Mortality From Analyses eTable 4. Associations of Grip Strength and Chair Stand Time According to Categories of Age eTable 5. Associations Between Muscle Strength and All-Cause Mortality When Time on Study (Follow-Up Time) or Age is Used as the Time Scale in the Cox Regression Model eFigure. Kaplan-Meier Survival Plots According to Categories of Grip Strength and Chair Stand Time

Supplement 2. Data Sharing Statement
