# Supplementary material for: Muscular Strength and Mortality in Women Aged 63 to 99 Years
Source: JAMA Netw Open. 2026 Feb 13;9(2):e2559367. doi: 10.1001/jamanetworkopen.2025.59367 (PMC12905654; doi:10.1001/jamanetworkopen.2025.59367)
Supplement: Supplement 1. — eTable 1. Baseline Characteristics by Vital Status eTable 2. Spearman Correlation Coefficients eTable 3. Results When Excluding Early Mortality From Analyses eTable 4. Associations of Grip Strength and Chair Stand Time According to Categories of Age eTable 5. Associations Between Muscle Strength and All-Cause Mortality When Time on Study (Follow-Up Time) or Age is Used as the Time Scale in the Cox Regression Model eFigure. Kaplan-Meier Survival Plots According to Categories of Grip Strength and Chair Stand Time [file jamanetwopen-e2559367-s001.pdf]

Supplemental Online Content

LaMonte MJ, Hyde ET, Nguyen S, et al. Muscular strength and mortality in women aged 63 to 99 years. *JAMA Netw Open*. 2026;9(2):e2559367. doi:10.1001/jamanetworkopen.2025.59367

eTable 1. Baseline Characteristics by Vital Status

eTable 2. Spearman Correlation Coefficients

eTable 3. Results When Excluding Early Mortality From Analyses

eTable 4. Associations of Grip Strength and Chair Stand Time According to Categories of Age

eTable 5. Associations Between Muscle Strength and All-Cause Mortality When Time on Study (Follow-Up Time) or Age is Used as the Time Scale in the Cox Regression Model

eFigure. Kaplan-Meier Survival Plots According to Categories of Grip Strength and Chair Stand Time

This supplemental material has been provided by the authors to give readers additional information about their work.

© 2026 LaMonte MJ et al. *JAMA Network Open*.

eTable 1. Baseline Characteristics by Vital Status

Characteristic	Vital Status		P-value[c]	Age-adjusted HR (95% CI)[d]
	**Alive** N = 3,508 **No. (%)**	**Dead** N = 1,964 **No. (%)**		
**Age, mean (SD), y**	76.5 (6.1)	82.4 (5.7)	<.001	2.09 (2.00, 2.19)
**Race-Ethnicity**			<.001	
White	1,391 (39.7)	1,315 (66.9)		1.00 (ref)
Black	1,393 (39.7)	458 (23.4)		0.85 (0.75, 0.95)
Hispanic/Latina	724 (20.6)	191 (9.7)		0.67 (0.57, 0.79)
**Education**				
≤High school	677 (19.3)	446 (22.7)	.012	1.00 (ref)
Some college	1,342 (38.3)	779 (39.7)		0.97 (0.86, 1.09)
≥College graduate	1,489 (42.4)	739 (37.6)		0.84 (0.74, 0.94)
**Current smoker**	73 (2.1)	62 (3.2)	.014	2.31 (1.79, 2.98)
**Use walking aid**	755 (21.5)	840 (42.7)	<.001	1.56 (1.43, 1.71)
**Self-rated health**			<.001	
Excellent/Very good	1,824 (52.0)	719 (36.6)		1.00 (ref)
Good	1,466 (41.7)	996 (50.7)		1.53 (1.39, 1.68)
Fair/Poor	218 (6.2)	249 (12.6)		2.72 (1.96, 2.96)
**Age at menopause, mean (SD), y**	47.9 (6.3)	48.5 (6.2)	.001	0.96 (0.92, 1.01)
**Height, mean (SD), cm**	160.1 (7.1)	159.0 (7.4)	<.001	1.01 (0.97, 1.05)
**Weight, mean (SD), kg**	72.9 (15.5)	70.1 (15.5)	<.001	1.08 (1.03, 1.14)
**BMI, mean (SD), kg·m^{-2}**	28.4 (5.7)	27.7 (5.7)	<.001	1.06 (1.02, 1.11)
**Estimated LBM, mean (SD), kg**	40.6 (6.3)	38.9 (6.3)	<.001	1.07 (1.02, 1.12)
**Systolic BP, mean (SD), mmHg**	124.9 (13.6)	127.3 (15.2)	<.001	1.07 (1.03, 1.12)
**Diastolic BP, mean (SD), mmHg**	72.8 (8.4)	72.1 (9.2)	.002	0.99 (0.95, 1.03)
**Comorbidities[a], mean (SD)**	1.4 (1.1)	1.9 (1.3)	<.001	1.24 (1.20, 1.28)
None	743 (21.2)	200 (10.2)	<.001	1.00 (ref)
1-2	2,213 (63.1)	1,182 (60.2)		1.55 (1.34, 1.81)
≥3	552 (15.7)	582 (29.6)		2.53 (2.00, 2.76)
**Log CRP, mean (SD), mg·dL^{-1}[e]**	0.60 (1.1)	0.67 (1.1)	.025	1.15 (1.09, 1.20)

© 2026 LaMonte MJ et al. *JAMA Network Open*.

**Alcohol, mean (SD), drinks·wk^{-1}**	0.9 (0.7)	0.8 (0.7)	<.01	0.93 (0.90, 0.95)
**Physical function[b], mean (SD)**	74.6 (23.3)	58.7 (26.7)	<.001	0.73 (0.70, 0.76)
**Light PA, mean (SD), min·d^{-1}**	297.1 (76.1)	264.5 (78.7)	<.001	0.76 (0.73, 0.80)
**MVPA, mean (SD), min·d^{-1}**	57.9 (35.4)	36.8 (28.0)	<.001	0.64 (0.60, 0.68)
**Sedentary time, mean (SD), hr·d^{-1}**	8.9 (1.6)	9.5 (1.6)	<.001	1.16 (1.11, 1.21)
**2.5-meter walk time, mean (SD), min**	7.1 (5.4)	8.5 (6.4)	<.001	1.17 (1.04, 1.21)
**Grip strength, mean (SD), kg**	20.4 (7.0)	17.1 (6.7)	<.001	---
**Chair stand time, mean (SD), sec**	14.9 (5.8)	16.2 (5.8)	<.001	---

[a]Comorbidities defined as heart disease, stroke, cancer, diabetes mellitus, hip fracture, osteoarthritis, depression, COPD, cognitive impairment, sensory impairment, frequent falls in past year.

[b]RAND-36 physical function score, range 0-100, higher score reflects better functional status.

[c]Based on student's t-test (continuous) or chi-squared test (categorical).

[d]For continuous variables the HR is per-1-SD unit defined using the SD in the survivors.

[e]n = 4,414.

© 2026 LaMonte MJ et al. *JAMA Network Open*.

eTable 2. Spearman Correlation Coefficients (N = 5,472)

Characteristic	Grip Strength	Chair Stand Time
Age	-0.35	0.11
Weight	0.19	0.15
BMI	0.08	0.12
Estimated LBM	0.26	0.13
Physical function	0.24	-0.30
Light PA	0.09	-0.14
MVPA	0.22	-0.25
Sedentary time	-0.09	0.11
2.5-meter walk time	-0.19	0.32
Chair stand time	-0.13	---

© 2026 LaMonte MJ et al. *JAMA Network Open*.

eTable 3. Results When Excluding Early Mortality From Analyses

	N (No. deaths)	Grip Strength HR (95% CI)	Chair Stands HR (95% CI)
Primary analysis	5,472 (1,964)	0.88 (0.84-0.93)	0.96 (0.93-1.01)
Excluding first 3-years	5,153 (1,645)	0.89 (0.84-0.93)	0.97 (0.92-1.01)
Excluding first 5-years	4,806 (1,298)	0.86 (0.81-0.92)	0.98 (0.92-1.03)

Hazard ratios are per 1-SD greater grip strength (7 kg) and faster chair stand time (6 sec).

Model adjusted for age, race and ethnicity, education, body weight, systolic and diastolic blood pressure, number of comorbidities, alcohol, age at menopause, use of walking aids, smoking, self-rated general health (model 2 covariates in the primary analysis, Table 2).

eTable 4. Associations of Grip Strength and Chair Stand Time According to Categories of Age

Age (years)	N (No. deaths)	Grip Strength HR (95% CI)	Chair Stands HR (95% CI)
<70	554 (58)	0.71 (0.54-0.93)	0.88 (0.71-1.08)
70-79	2,226 (441)	0.89 (0.81-0.98)	0.93 (0.86-1.02)
80-89	2,469 (1,292)	0.89 (0.84-0.95)	0.97 (0.91-1.02)
≥90	223 (173)	0.90 (0.74-1.10)	1.17 (1.00-1.36)

Hazard ratios are for a 1-SD unit greater grip strength (7 kg) and faster chair stand time (6 sec).

Model adjusted for age, race and ethnicity, education, body weight, systolic and diastolic blood pressure, number of comorbidities, alcohol, age at menopause, use of walking aids, smoking, self-rated general health.

© 2026 LaMonte MJ et al. *JAMA Network Open*.

eTable 5. Associations Between Muscle Strength and All-Cause Mortality When Time on Study (Follow-Up Time) or Age is Used as the Time Scale in the Cox Regression Model

	**Grip Strength (kg)**				**P-Trend**	**Per 1-SD**
	**<14**	**14-19**	**20-24**	**>24**		
N	1,085	1,632	1,280	1,475		
No. deaths (rate)	554 (67.2)	695 (52.3)	405 (37.4)	310 (23.5)		
Time Scale:						
Time on Study	1.00 (ref)	0.94 (0.85-1.06)	0.85 (0.75-0.97)	0.67 (0.58-0.78)	<.001	0.88 (0.84-0.93)
Age	1.00 (ref)	0.95 (0.85-1.07)	0.85 (0.74-0.97)	0.66 (0.57-0.77)	<.001	0.89 (0.83-0.92)
	**Chair Stand Time (sec)**					
	≥16.7 (slower)	16.6-13.7	13.6-11.2	≤11.1 (faster)		
N	2,076	1,304	1,077	1,015		
No. deaths (rate)	975 (60.2)	435 (39.1)	316 (33.9)	238 (26.5)		
Time Scale:						
Time on Study	1.00 (ref)	0.79 (0.69-0.88)	0.76 (0.67-0.87)	0.63 (0.54-0.73)	<.001	0.96 (0.93-1.01)
Age	1.00 (ref)	0.83 (0.74-0.93)	0.81 (0.72-0.93)	0.67 (0.58-0.78)	<.001	0.99 (0.94-1.03)

The age time scale was defined by age at entry (OPACH baseline) and age at end (death or censoring at end follow-up).

Death rate is per 1,000 person-years.

Model adjusted for race and ethnicity, education, body weight, systolic and diastolic blood pressure, number of comorbidities, alcohol, age at menopause, use of walking aids, smoking, self-rated general health (model 2 covariates in the primary analysis, Table 2).

© 2026 LaMonte MJ et al. *JAMA Network Open*.

eFigure. Kaplan-Meier Survival Plots According to Categories of Grip Strength and Chair Stand Time

Panel A. Grip Strength

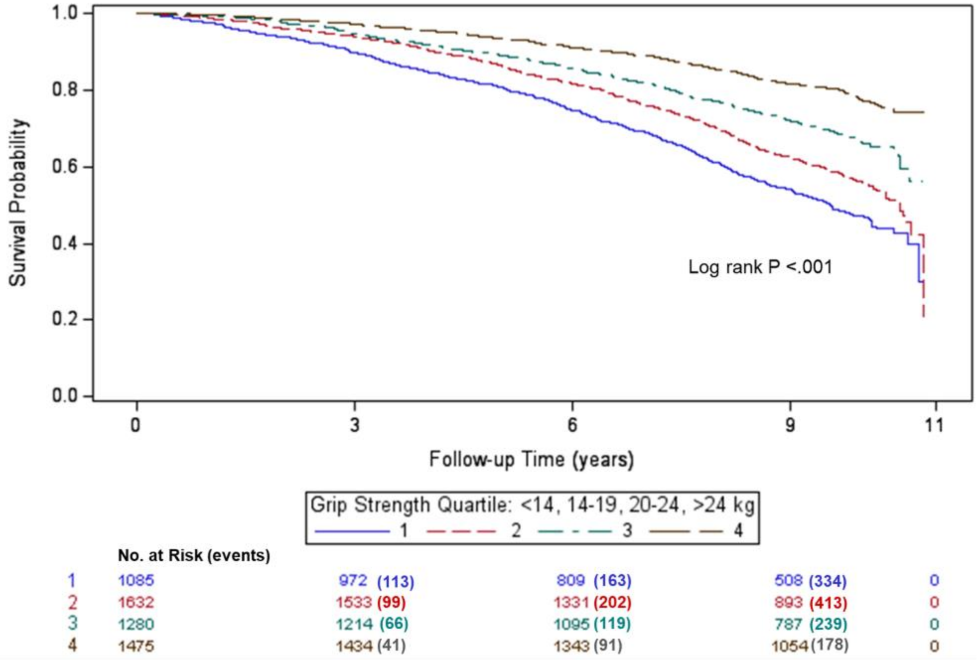

Panel B. Chair Stand Time

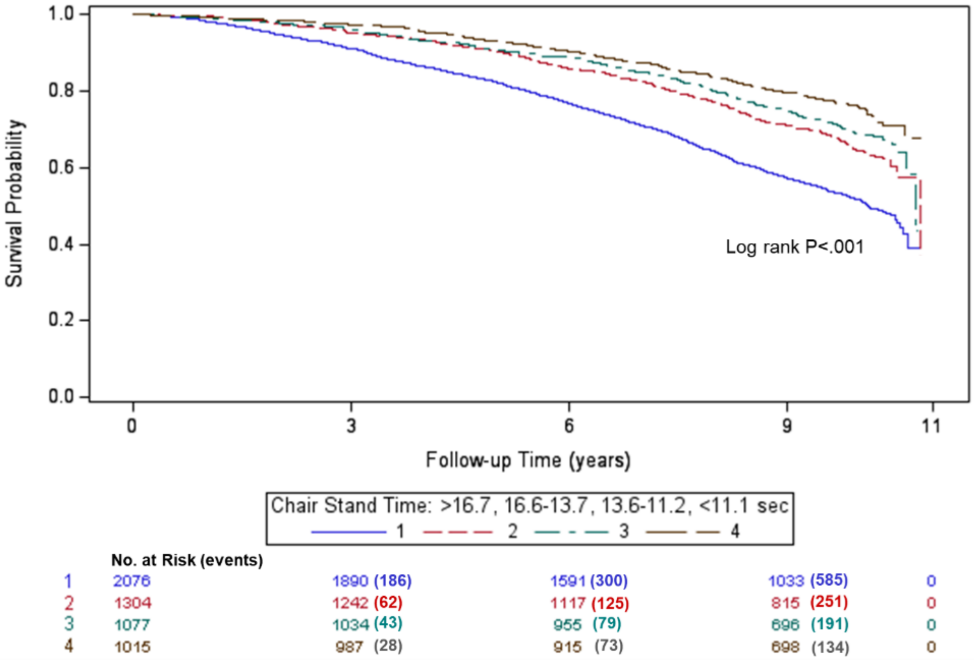

© 2026 LaMonte MJ et al. *JAMA Network Open*.
