# Supplementary material for: Muscular Strength and Mortality in Women Aged 63 to 99 Years
Source: JAMA Netw Open. 2026 Feb 13;9(2):e2559367. doi: 10.1001/jamanetworkopen.2025.59367 (PMC12905654; doi:10.1001/jamanetworkopen.2025.59367)
Supplement: Supplement 2. — Data Sharing Statement [file jamanetwopen-e2559367-s002.pdf]

Data Sharing Statement

LaMonte. Muscular Strength and Mortality in Women Aged 63 to 99 Years. *JAMA Netw Open*. Published February 13, 2026. doi:10.1001/jamanetworkopen.2025.59367

Data
Data available: Yes
Data types: Other (please specify)
Additional Information: Data will be made available upon reasonable request to the corresponding author and with approval pursuant to policies of the Women's Health Initiative.
How to access data: Data will be made available upon reasonable request to the corresponding author and with approval pursuant to policies of the Women's Health Initiative.
When available: With publication

Supporting Documents
Document types: None

Additional Information
Who can access the data: Individuals who receive approval for data use by the Women's Health Initiative.
Types of analyses: Data availability will be based on the nature of the requested use with sufficient justification.
Mechanisms of data availability: An approved paper proposal and signed data use agreement through the Women's Health Initiative is required.
Any additional restrictions: All conditions stipulated by the Women's Health Initiative data use and publications policies and procedures must be satisfied in order to gain access to the data.
